# Evaluation of the intestinal permeability of rosemary (*Rosmarinus officinalis* L.) extract polyphenols and terpenoids in Caco-2 cell monolayers

**DOI:** 10.1371/journal.pone.0172063

**Published:** 2017-02-24

**Authors:** Almudena Pérez-Sánchez, Isabel Borrás-Linares, Enrique Barrajón-Catalán, David Arráez-Román, Isabel González-Álvarez, Elena Ibáñez, Antonio Segura-Carretero, Marival Bermejo, Vicente Micol

**Affiliations:** 1 Institute of Molecular and Cell Biology, Miguel Hernández University, Avda. Universidad s/n, Elche, Spain; 2 Department of Analytical Chemistry, Faculty of Sciences, University of Granada, Avda Fuentenueva s/n, Granada, Spain; 3 Research and Development of Functional Food Centre (CIDAF), Health Science Technological Park, Avda. del Conocimiento n° 37, Armilla, Spain; 4 Pharmacokinetics and Pharmaceutical Technology Area, Engineering Department, Universidad Miguel Hernández, San Juan de Alicante, Alicante, Spain; 5 INVTROTECNIA S.L., Santiago Grisolía 2, Tres Cantos, Madrid, Spain; 6 Laboratory of Foodomics, Institute of Food Science Research-CIAL (CSIC-UAM), Nicolás Cabrera 9, Campus Cantoblanco, Madrid, Spain; 7 CIBER, Fisiopatología de la Obesidad y la Nutrición, CIBERobn, Instituto de Salud Carlos III (CB12/03/30038), Spain; Universidad de Malaga, SPAIN

## Abstract

Rosemary (*Rosmarinus officinalis*) is grown throughout the world and is widely used as a medicinal herb and to season and preserve food. Rosemary polyphenols and terpenoids have attracted great interest due to their potential health benefits. However, complete information regarding their absorption and bioavailability in Caco-2 cell model is scarce. The permeation properties of the bioactive compounds (flavonoids, diterpenes, triterpenes and phenylpropanoids) of a rosemary extract (RE), obtained by supercritical fluid extraction, was studied in Caco-2 cell monolayer model, both in a free form or liposomed. Compounds were identified and quantitated by liquid chromatography coupled to quadrupole time-of-flight with electrospray ionization mass spectrometry analysis (HPLC-ESI-QTOF-MS), and the apparent permeability values (P_app_) were determined, for the first time in the extract, for 24 compounds in both directions across cell monolayer. For some compounds, such as triterpenoids and some flavonoids, P_app_ values found were reported for the first time in Caco-2 cells.Our results indicate that most compounds are scarcely absorbed, and passive diffusion is suggested to be the primary mechanism of absorption. The use of liposomes to vehiculize the extract resulted in reduced permeability for most compounds. Finally, the biopharmaceutical classification (BCS) of all the compounds was achieved according to their permeability and solubility data for bioequivalence purposes. BCS study reveal that most of the RE compounds could be classified as classes III and IV (low permeability); therefore, RE itself should also be classified into this category.

## Introduction

Rosemary (*Rosmarinus officinalis* L.) is a shrub from the *Labiatae* (*Lamiaceae*) family that is primarily distributed throughout the Mediterranean area. It has been demonstrated that rosemary and its major compounds, the diterpenes carnosic acid (CA) and carnosol (CAR) and the caffeoyl derivative rosmarinic acid, exert various beneficial effects on health, including potent antioxidant capacity [1, 2] and hepaprotective [3], antimicrobial [4, 5], anti-inflammatory [6, 7], anti-cancer [8–13] and antidiabetic effects [14].

We have previously reported the antiproliferative effects of a polyphenol-enriched rosemary extract (RE) obtained via supercritical extraction in several colon cancer cell models [13]. Transcriptomic and metabolomic analysis suggested that the extract activated genes related to antioxidant phase II enzymes and cell cycle progression [12]. This result was consistent with the activation of nuclear receptor factor 2 (NRF2)-dependent pathways, ROS and glutathione metabolism by CA as determined using a comprehensive Foodomics approach [11]. However, bioguided purification of the extract revealed potential synergistic antiproliferative effects between diterpenes and triterpenes [13].

The rosemary polyphenols (di- and triterpenes) are considered promising drug candidates in the pharmaceutical, cosmetic, and nutritional fields. However, these compounds exhibit physicochemical characteristics that result in unfavorable transcellular transport across epithelial barriers. Several attempts have been made to evaluate the absorption of the active compounds from rosemary extract in cell and animal models. The bioavailability of major diterpenoids derived from rosemary extract in different tissues in a rat model of obesity has been reported [15]. Moreover, the absorption, distribution and elimination of carnosic acid have been evaluated in rats [16]. However, complete information regarding the absorption and bioavailability of a full range of rosemary bioactive compounds (flavonoids, diterpenes and triterpenes) in Caco-2 cell monolayers is not available. Studies examining the permeation of carnosic acid and carnosol in Caco-2 cells are present in the literature, but permeation was either insufficiently characterized [17] or was found to be almost negligible [18]. In addition, no comparative data regarding the permeation and potential absorption mechanisms of all compounds present in rosemary extract have been generated so far.

Liposomes have been utilized to improve solubility and selectivity and to improve bioavailability of poorly soluble drugs by modifying drug absorption, reducing metabolism, prolonging biological half-life or reducing toxicity. Phospholipid bilayers of liposomes are very similar to the structure of cell membranes; therefore, liposomes can deliver encapsulated drugs, peptides and natural compounds by fusing with target cell membranes, and their specificity can be improved by antibody coupling [19–22]. Several plant polyphenols and bioactive compounds, such as catechins [23], anthraquinones [24], phenylpropanoids [25], and diterpenes [1], have been shown to possess the capacity to interact with phospholipid membranes.

In the present paper, a Caco-2 cell monolayer model, which is a well-accepted model of human intestinal absorption [26], was used to comprehensively study and compare the permeation properties of different bioactive compounds (flavonoids, diterpenes, triterpenes and phenylpropanoids) identified in a rosemary extract (RE) by HPLC-ESI-QTOF-MS and obtained via supercritical fluid extraction [27]. The permeation behaviors of the compounds were compared when the extract was in a free form or encapsulated into liposomes. Moreover, all compounds were biopharmaceutically classified based on their permeability and solubility data for bioequivalence purposes.

## Materials and methods

### Chemicals and cell culture materials

All chemicals were of analytical reagent grade and were used as received. For mobile phase preparation, formic acid and acetonitrile were purchased from Sigma-Aldrich (Steinheim, Germany) and Fisher Scientific (Madrid, Spain), respectively. Water was purified using a Milli-Q system from Millipore (Bedford, MA, USA). The standard compounds ursolic acid, rosmarinic acid, genkwanin, diosmetin and luteolin were obtained from Extrasynthese (Genay, France). Carnosol, carnosic acid and apigenin were obtained from Fluka, Sigma-Aldrich (Steinheim, Germany). Egg yolk phosphatidylcholine was purchased from Lipoid GmbH (Ludwigshafen, Germany), and cholesterol was obtained from Avanti Polar Lipids (Alabaster, AL, USA). Methanol and dimethyl sulfoxide (DMSO) from Fisher Scientific (Madrid, Spain) were used to prepare the stock solutions utilized for quantitation purposes. Hank´s balanced culture medium (HBSS), Dulbecco´s Modified Eagle’s Medium (DMEM), fetal bovine serum (FBS), 100 U/mL penicillin/streptomycin, MEM Non-Essential Amino Acids Solution (100x) and 1 M HEPES were obtained from Gibco/Thermo Fisher Scientific (Waltham, MA, USA). The human colon adenocarcinoma cell line Caco-2 was obtained from the American Type Culture Collection. Caco-2 cells were cultured in DMEM containing D-glucose (4.5 g/L) and supplemented with 10% FBS, 1% NEAA, 1% HEPES, penicillin (100 U/mL) and streptomycin (100 μg/mL) at 37°C in a humidified atmosphere with 5% CO_2_.

### Cell viability assay

The cytotoxic effects of free and encapsulated RE extract on Caco-2 cells were tested using the 3-(4,5-dimethylthiazol-2-yl)-2,5-diphenyltetrazolium bromide (MTT) assay. Caco-2 cells were plated in 96-well plates (Costar, Fisher Scientific, Pittsburgh, PA, USA) until cell monolayers were obtained. Then, the medium was aspirated, and cells were treated with different concentrations of RE for 2 h. The medium was removed, and cells were washed with PBS and incubated with MTT for 3–4 h at 37°C and 5% CO_2_. The medium was removed, and 100 μL of DMSO per well was added to dissolve the formazan crystals. The plates were shaken for 15 min, and absorbance was measured using a microplate reader (SPECTROstar Omega, BMG LabTech GmbH, Germany) at 570 nm.

### Caco-2 monolayer transport studies

Caco-2 cells are a well-established *in vitro* model for the investigation of intestinal permeabilities of different compounds or drugs [28–30]. Cells were seeded at a density of 5.0 x 10^5^ cells on 6-well transwell polycarbonate filters (Millipore, Spain). Cell culture was maintained at 37°C under 90% humidity and 5% CO_2_. The medium was replaced every 2–3 days for both the apical (AP) and basal (BL) sides of the transwell filters. Cell monolayers were used 19–21 days after seeding, once confluence and differentiation were achieved. The integrity of each cell monolayer was checked by measuring the trans-epithelial electrical resistance (TEER) before and after the experiments with an epithelial voltohmmeter (Millicell-ERS®) (see results on S3 Table). Permeability studies were performed by adding the RE at a concentration of 200 μg/mL or the liposomal RE formulation.

The liposomal formulation was prepared using the conventional thin film hydration technique. Egg yolk phosphatidylcholine and cholesterol (80:20 w/w) and 10% (w/w) RE with respect to total phospholipids were dissolved in a 1:1 mixture of chloroform/methanol. A lipid film was obtained by evaporating the organic solvent under a stream of nitrogen (N_2_) and then further vacuum-dried for 3–4 h to remove any residual organic solvent. The film was hydrated with HEPES buffer (100 mM NaCl, 0.1 mM EDTA, 10 mM HEPES, pH 7.4) via vigorous vortexing for 30 min at 37°C. The multilamellar liposomal suspension was filter-extruded through a 100-nm polycarbonate Track-Etch Nuclepore membrane (Whatman, UK) to obtain large unilamellar vesicles (LUVs). Size reduction was carried out with 15 extrusion cycles performed by hand with a LiposofastTM syringe extruder (Avestin Inc., Canada). The resulting suspension was centrifuged at 4,000 rpm for 30 min (2 cycle) using an Amicon® Ultra (Millipore, Hayward, CA, USA) to separate the liposomes from non-encapsulated drug. The liposomal suspension was diluted to a concentration of 1.5 mM with HBSS for absorption experiments in the receiving chamber.

The transport experiment was initiated by removing the culture medium from the AP and BL sides of the transwell filters. The Caco-2 monolayers were rinsed twice with pre-warmed HBSS medium (pH 7.4) and incubated with the same solution at 37°C for 30 min. The test compounds were added to the AP (2.2 mL) or BL side (3.2 mL), while the receiving chamber contained the corresponding volume of HBSS. Incubation was performed at 37°C for 120 min, with shaking at 50 rpm.

To follow transport across the cell monolayer, several culture medium samples of 0.2 mL were collected at different time points (0, 30, 60, 90 and 120 min) from the AP or BL sides during the permeability assay. The volume of the samples taken at each time point was replaced with the same volume of HBSS to maintain the total volume in the chamber throughout the experiment.

Before HPLC-ESI-QTOF-MS analysis, samples were centrifuged for 15 min at 12,000 rpm and 4°C. The supernatant was spiked with 5 μg/mL of an internal standard (luteolin) to ensure the reproducibility of the results between analyses, and samples were stored at -80°C until analysis was complete. At the end of the transport study, the Caco-2 cell monolayers were also collected, and the cells were lysed with 3 subsequent freeze-thaw cycles (10 min each) followed by bath sonication. The samples were centrifuged for 15 min at 14,000 rpm and 4°C, and the supernatants (cytoplasmic fraction) and the pellets (cell membranes) were spiked with 5 μg/mL luteolin as an internal standard. Then, the samples were subjected to protein precipitation using methanol, vortex-mixed, maintained at -20°C for 2 h and centrifuged at 12,000 rpm for 15 min at 4°C. Finally, the supernatants were evaporated in a vacuum concentrator, re-dissolved in 100 μL of HBSS culture medium and stored at -80°C until analysis was performed.

Apparent permeability values (P_app_) for each compound were calculated according to the following equation:

$$Papp=\frac{dQ}{dt}\cdot \frac{1}{A\cdot Co\cdot 60}$$

where P_app_ is the apparent permeability (cm/s), dQ/dt is the steady state flux, A is the diffusion area of the monolayers (cm^2^), C_0_ is the initial concentration of the drug in the donor compartment (μM) and 60 is a conversion factor [31].

### Analytical methodology

HPLC analyses were performed on an Agilent 1260 HPLC instrument (Agilent Technologies, Palo Alto, CA, USA) equipped with a binary pump, an online degasser, an auto-sampler, a thermostatically controlled column compartment, and diode array detectors. The samples were separated on an Agilent Zorbax Eclipse Plus C18 column (1.8 μm, 4.6 × 150 mm). The mobile phases consisted of water plus 0.1% formic acid as mobile phase A and acetonitrile as mobile phase B, using a gradient elution based on the following profile: 0 min, 5% B; 5 min, 62% B; 10 min, 68% B; 19 min, 80% B; 34 min, 95% B; 37 min, 5% B and finally a conditioning cycle of 5 min under the initial conditions before the next analysis. The flow rate was 0.8 mL/min, the column temperature set at 25°C and the auto-sampler compartment was refrigerated at 4°C to avoid sample degradation.

Detection was performed using an Agilent 6540 Ultra High Definition (UHD) Accurate-Mass Q-TOF mass spectrometer equipped with a Jet Stream dual ESI interface, which increases LC/MS sensitivity by improving the spatial focusing of electrospray droplets. Mass spectra were recorded in negative ionization mode over a mass range from 100–1700 m/z. Ultrahigh pure nitrogen was used as the drying and nebulizing gas. The operating parameters were as follows: drying gas flow rate, 10 L/min; drying gas temperature, 325°C; sheath gas temperature, 400°C; sheath gas flow, 12 L/min; nebulizer, 20 psig; capillary, 4000 V; fragmentor, 130 V, nozzle voltage, 500 V; skimmer, 45 V and octopole 1 RF Vpp, 750 V. Continuous infusion of the reference ions m/z 112.985587 (trifluoroacetate anion) and 1033.988109 (adduct of hexakis (^1^H,^1^H, ^3^H-tetrafluoropropoxy) phosphazine or HP-921) in negative ion mode were used to correct each spectrum to achieve accurate mass measurements, typically better than 2 ppm. All operations, acquisition and analysis of data were controlled using Masshunter workstation software version B.06.00 (Agilent Technologies, USA).

### Statistical analysis

Two-way ANOVA and statistical comparisons of the different treatments were performed using Tukey´s post-test in GraphPad Prism version 5.00 (GraphPad Software, San Diego, CA, USA). Differences were considered statistically significant at p < 0.05. Statistical significance is detailed in figures using the following symbols: * *p*<0.05, ** *p*<0.01 and *** *p*<0.001.

## Results and discussion

We recently reported the composition of the rosemary leaf extract (RE) under study as determined by HPLC-ESI-QTOF-MS analysis [13]. In that study, bioguided purification of the most active fractions was undertaken to identify the compounds bearing the highest antiproliferative capacities in a colon cancer cell model. The aim of the present study was to compare the intestinal absorption and permeability behavior of all compounds in the RE, both when the extract was in a free or in an encapsulated formulation, using the Caco-2 model system. To achieve this, the quantitation of bioactive compounds and absorption results will be discussed by grouping the different bioactive compounds into families (flavonoids, diterpenes, triterpenes and phenylpropanoid derivatives).

### HPLC ESI-UHD-Qq-TOF-MS analysis

For quantitative purposes, six standard calibration graphs were prepared using HBSS culture medium to quantify the major compounds in the rosemary extract using the following commercial standards: carnosol, carnosic acid, ursolic acid, rosmarinic acid, genkwanin, and diosmetin.

Variability of peak area produced by mass data change over time may be a limitation for quantification by mass spectrometry, depending on the instrument. Thus, an internal standard (luteolin) was used at a concentration of 5 μg/mL to improve reproducibility. This compound met all requirements in terms of structural similarity compared with the analytes, retention time (which did not interfere with other compounds) and compensation for potential variations in instrumental analysis. Moreover, to avoid ion suppression or signal enhancement of signals due to interference derived from the matrix, the calibration solutions were prepared in HBSS culture medium, which was the same matrix containing the samples in the permeability assay. Therefore, compound concentrations were determined using the corrected area for each individual compound (three replicates) and by interpolating the corresponding calibration curve. The limit of detection (LOD) ranged from 0.0008 ± 0.0002 (diosmetin) to 0.04 ± 0.01 (ursolic acid) μg/mL. The limit of quantification (LOQ) ranged from 0.003 ± 0.001 (diosmetin) to 0.14 ± 0.03 (ursolic acid) μg/mL.

Quantification was performed using available commercial standards for carnosol, diosmetin, apigenin, genkwanin and carnosic, ursolic and rosmarinic acids. Compounds that had no commercially available standards were tentatively quantified on the basis of calibration curves for other compounds with similar structures. Thus, rosmanol, its isomers epiisorosmanol and epirosmanol, miltipolone, rosmadial and rosmaridiphenol were quantified with the carnosol standard. Hinokione and 12-methoxycarnosic acid were expressed as carnosic acid. The ursolic acid calibration curve was used to quantify augustic, benthamic, micromeric and betulinic acids, in addition to anemosapogenin. Finally, the genkwanin standard was used for cirsimaritin quantification; [9]-shogaol was expressed as rosmarinic acid, and diosmetin was used to estimate the hispidulin content.

### Determination of P_app_ values

After quantifying the concentrations of all identified compounds in samples derived from the apical and basolateral compartments, as described in the previous section, concentration values were used to obtain P_app_ values based on the formula described in the Materials section. Values are shown in Table 1 for both the AB and BA directions and for free and encapsulated formulations. Some compounds could not be quantified because their concentrations fell below LOQ values, sometimes for both directions or formulations (free or encapsulated) and occasionally for one of these. Detailed explanations for clustering compounds in families are provided in the following sections.

**Table 1:** P_app_ values.

		Non encapsulated RE				Encapsulated RE			
		AB direction		BA direction		AB direction		BA direction	
Compound	Family	P_app_	SD	P_app_	SD	P_app_	SD	P_app_	SD
Apigenin	Flavonoid	N.C.		N.C.		N.C.		N.C.	
Cirsimaritin	Flavonoid	3.33E-05	1.17E-06	3.07E-05	6.29E-06	N.A.	N.A.	N.A.	N.A.
Diosmetin	Flavonoid	2.50E-06	N.E.	4.13E-06	1.38E-06	N.A.	N.A.	N.A.	N.A.
Hispidulin	Flavonoid	9.05E-06	3.04E-06	1.31E-05	9.60E-07	N.A.	N.A.	N.A.	N.A.
Genkwanin	Flavonoid	1.49E-05	2.47E-06	1.62E-05	3.42E-06	N.A.	N.A.	N.A.	N.A.
Carnosol	Diterpene	1.46E-05	2.10E-06	1.35E-05	5.15E-06	4.03E-08	3.18E-08	2.61E-07	2.72E-08
Carnosol isomer	Diterpene	2.15E-05	3.30E-06	2.13E-05	5.73E-06	4.03E-08	3.18E-08	4.03E-08	3.18E-08
Carnosic acid	Diterpene	1.27E-04	1.55E-05	9.04E-05	1.09E-05	N.A.	N.A.	N.A.	N.A.
12-Methoxycarnosic acid	Diterpene	1.85E-05	4.04E-06	2.77E-05	5.12E-06	N.A.	N.A.	N.A.	N.A.
Rosmadial	Diterpene	1.90E-05	2.84E-06	1.59E-05	1.20E-06	2.67E-06	6.43E-07	1.24E-05	9.19E-07
Rosmanol	Diterpene	4.59E-05	7.48E-06	4.42E-05	3.53E-06	2.91E-05	9.45E-06	3.33E-05	N.C.
Epirosmanol	Diterpene	5.51E-05	2.79E-05	1.21E-05	7.90E-06	1.92E-06	4.17E-07	2.63E-06	6.02E-07
Epiisorosmanol	Diterpene	1.04E-04	1.79E-05	9.23E-06	6.55E-06	8.18E-06	3.02E-06	1.30E-05	3.52E-06
Miltipolone	Diterpene	1.76E-05	1.97E-06	N.A.	N.A.	N.A.	N.A.	1.68E-07	8.91E-08
Hinokione	Diterpene	N.A.	N.A.	1.36E-05	1.22E-06	N.A.	N.A.	N.A.	N.A.
Rosmaridiphenol	Diterpene	2.43E-06	6.44E-07	2.68E-06	1.28E-06	N.A.	N.A.	N.A.	N.A.
Augustic acid	Triterpene	7.35E-06	3.64E-06	7.93E-06	4.12E-06	N.A.	N.A.	N.A.	N.A.
Betulinic acid	Triterpene	0.00E+00	0.00E+00	6.30E-06	5.66E-07	N.A.	N.A.	N.A.	N.A.
Anemosapogenin	Triterpene	3.27E-06	1.79E-06	6.17E-06	2.99E-06	N.A.	N.A.	N.A.	N.A.
Micromeric acid	Triterpene	7.20E-06	2.26E-06	2.45E-06	1.63E-06	N.A.	N.A.	N.A.	N.A.
Benthamic acid	Triterpene	5.20E-06	2.40E-06	7.03E-06	3.25E-06	N.A.	N.A.	N.A.	N.A.
Ursolic acid	Triterpene	0.00E+00	0.00E+00	7.20E-06	2.26E-06	N.A.	N.A.	N.A.	N.A.
(9)-Shogaol	Phenylpropanoid	N.C.		N.C.		N.C.		N.C.	
(9)-Shogaol Isomer	Phenylpropanoid	N.C.		N.C.		N.C.		N.C.	

P_app_ values in cm/s for all the compounds quantitated in the free and encapsulated formulations of RE in both AB and BA directions. Values represent the mean of six independent replicates ± standard deviation (SD). N.C., non-calculated (see text for further explanations). N.E., no error was obtained as only one of the replicates was used as the other presented values below LOQ. N.A. indicates P_app_ = 0 (non-absorbed).

#### Flavonoids and phenylpropanoid derivatives

**Fig 1:**
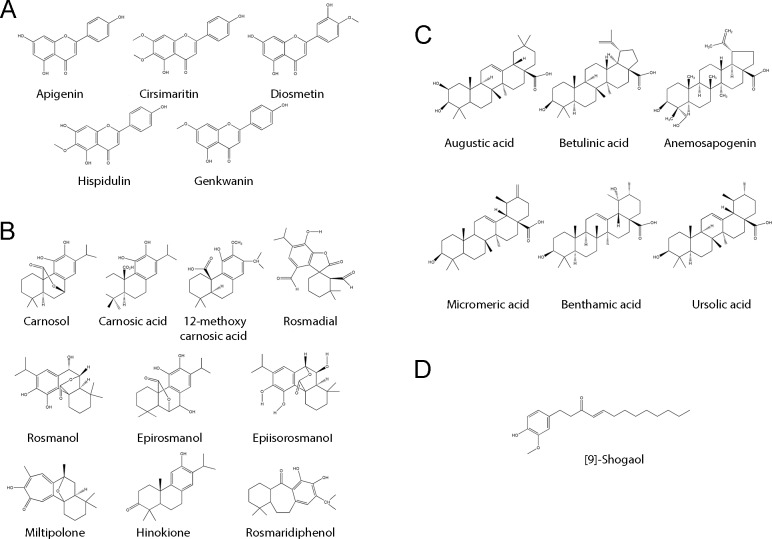
Chemical structures of all the compounds identified in RE. A: Flavonoids, B: diterpenes, C: triterpenes, D: phenylpropanoid derivatives.

The phenylpropanoid derivative (9)-shogaol (a gingerol-like compound) and its isomer (Fig 1D) were present in all the samples but were below their respective LOQs; thus, no conclusions could be drawn for these compounds. A deeper analysis of the samples revealed that both compounds were partly retained by Caco-2 cells without any transport to the receptor chamber because amounts of these compounds below their LOQs were also detected in the cytoplasmic fractions of Caco-2 cells, as previously reported for (6)-shogaol [32].

Despite the fact that flavonoids were less frequently represented in the extract based on our previous quantitative characterization [13], their potential absorption and biological activity are worthy of analysis. Flavonoids present in the RE were apigenin, hispidulin, diosmetin, genkwanin and cirsimaritin (Fig 1A). Conclusions regarding apigenin in the context of the whole extract could not be reached because the concentration fell below its LOQ. However, several studies have reported apigenin absorption in the Caco-2 cell model [33–37]. For the other four flavonoids, no permeation was detected in either of the directions when the RE sample was encapsulated into liposomes. However, significant permeation was detected for the RE in its free form in both the AB and BA directions. The highest values for AB were obtained for cirsimaritin and genkwanin, with no significant differences between them (*p*>0.05); this is the first report of cirsimaritin and genkwanin values in Caco-2 cells. Diosmetin and hispidulin exhibited lower permeation values that demonstrated statistically significant differences compared to cirsimaritin (*p*<0.001 and 0.01, respectively), but there was no difference between them (*p*>0.05). Higher values than those observed in our study have been reported for pure diosmetin in both flux directions [38], but it must be noted that competition with other polyphenols and terpenoids may take place in the whole extract. Absorption values for hispidulin and apigenin in the whole extract were in agreement with those reported for the pure compounds [39, 40]. Regarding the basal-apical direction, cirsimaritin, genkwanin and hispidulin demonstrated similar permeation values with no significant differences between them (*p*>0.05), followed by diosmetin, which had the lowest value (*p*<0.001).

Based on our results, we can conclude that rosemary flavonoids did not exhibit differences when comparing the two flux directions (AB and BA) for the free-form extract, which suggests a passive diffusion transport mechanism. No transport at all was observed when these compounds were incorporated into liposomes.

#### Diterpenoids

Diterpenoids (10.87% w/w of the extract) together with triterpenoids constitute the major group of compounds in the extract and comprise up to 10 compounds (Fig 1B and Table 1) [13]. The diterpenoids present in the RE were carnosic acid, carnosol, rosmanol and its isomers (epiisorosmanol and epirosmanol), 12-methoxycarnosic acid, rosmadial, rosmaridiphenol, hinokione and miltipolone.

Among all the diterpenes and the non-encapsulated RE, the highest permeability in the AB direction was obtained for carnosic acid (with *p*<0.001), followed by epirosmanol and rosmanol, which did not demonstrate statistically significant differences between them (*p>0*.*05*). A carnosol isomer, rosmadial, 12-methoxy carnosic acid, miltipolone and carnosol exhibited Papp values ranging from 2.1 to 1.46E-05. In contrast, the diterpenoid rosmaridiphenol demonstrated lower absorption (P_app_ in the E-06 range), while hinokione showed no absorption. For the BA direction, the highest permeability value was obtained for carnosic acid (*p*<0.001) followed by the rest of the compounds, with no relevant differences between them (*p>*0.05) with the exception of miltipolone, which was not absorbed.

In general, absorption values of the compounds were lower for the encapsulated form than for the free extract. When absorption for the encapsulated form in the AB direction was studied, rosmanol demonstrated the highest permeability followed by epiisorosmanol, rosmadial and epirosmanol. The encapsulated formulation dramatically decreased the permeability for epirosmanol, epiisorosmanol (both at *p*<0.001) and rosmanol (*p*<0.05) in the AB direction, with no changes in the BA direction compared with the free formulation. As there is no difference in polarity between these compounds (see Log P in Table 2), it can be assumed that the few differences in their structures must be crucial for their absorption. Both isomers of carnosol showed significantly lower absorption values than those previously reported. The rest of the compounds showed no absorption in the encapsulated extract (12-methoxy carnosic acid, carnosic acid, rosmaridiphenol, miltipolone and hinokione). For the BA direction, identical behavior for the abovementioned compounds was observed.

**Table 2:** Chemical family, physicochemical and permeation data and BCS classification.

Compound	Family	D (M)	S (mg/mL)	Vs	D_0_	Solubility	Log P	Permeability	BCS Class
								Based on Log P	Based on Log P
								Based on P_app_	Based on P_app_
Apigenin	Flavonoid	3.70E-02	4.47E-03	8.28	3.31E-02	High	1.90	Low	III
								N.C.	N.C.
Cirsimaritin	Flavonoid	3.18E-02	2.57E-04	123.79	4.95E-01	High	2.04	Low	III
								Low	III
Diosmetin	Flavonoid	3.33E-02	4.27E-03	7.81	3.12E-02	High	1.78	Low	III
								Low	III
Hispidulin	Flavonoid	3.33E-02	1.58E-03	21.01	8.41E-02	High	1.78	Low	III
								Low	III
Genkwanin	Flavonoid	3.52E-02	4.17E-04	84.39	3.38E-01	High	2.17	Low	III
								Low	III
Carnosol	Diterpene	3.03E-02	2.57E-05	1177.43	4.71	Low	4.58	High	II
								Low	IV
Carnosol Isomer	Diterpene	3.03E-02	2.57E-05	1177.43	4.71	Low	4.58	High	II
								Low	IV
Carnosic acid	Diterpene	3.01E-02	1.51E-02	1.99	7.95E-03	High	5.14	High	I
								Low	III
12-methoxycarnosic acid	Diterpene	2.89E-02	5.50E-03	5.25	2.10E-02	High	5.4	High	I
								Low	III
Rosmadial	Diterpene	2.90E-02	9.12E-05	318.37	1.27	Low	3.74	High	II
								Low	IV
Rosmanol	Diterpene	2.89E-02	5.75E-05	501.65	2.01	Low	3.70	High	II
								Low	IV
Epirosmanol	Diterpene	2.89E-02	5.75E-05	501.65	2.01	Low	3.70	High	II
								Low	IV
Epiisorosmanol	Diterpene	2.89E-02	5.75E-05	501.65	2.01	Low	3.70	High	II
								High	II
Miltipolone	Diterpene	3.33E-02	3.55E-05	938.23	3.75	Low	1.02	Low	IV
								Low	IV
Hinokione	Diterpene	3.33E-02	2.19E-05	1521.42	6.09	Low	5.85	High	II
								N.C.	N.C.
Rosmaridiphenol	Diterpene	3.16E-02	1.55E-06	20404.08	81.62	Low	4.89	High	II
								Low	IV
Augustic acid	Triterpene	2.11E-02	1.15E-04	184.25	7.37E-01	High	6.52	High	I
								Low	III
Betulinic acid	Triterpene	2.19E-02	2.04E-05	1072.43	4.29	Low	7.38	High	II
								N.C.	N.C.
Anemosapogenin	Triterpene	2.12E-02	1.20E-04	175.96	7.04E-01	High	6.32	High	I
								Low	III
Micromeric acid	Triterpene	2.20E-02	1.23E-04	178.77	7.15E-01	High	6.91	High	I
								Low	III
Benthamic acid	Triterpene	2.12E-02	5.89E-04	35.93	1.44E-01	High	6.13	High	I
								Low	III
Ursolic acid	Triterpene	2.19E-02	1.51E-05	1446.66	5.79	Low	7.33	High	II
								N.C.	N.C.
[9]-Shogaol	Phenylpropanoid	3.14E-02	1.29E-06	24375.88	97.50	Low	5.26	High	II
								N.C.	N.C.
[9]-Shogaol Isomer	Phenylpropanoid	3.14E-02	1.29E-06	24375.88	97.50	Low	5.26	High	II
								N.C.	N.C.

Chemical family, physicochemical and permeation data and BCS classification for all the compounds of RE studied in the absorption assay in the free form and considering 10 mg dose scenario.

It can be concluded that among all the diterpenes, rosmanol and its isomers epiisorosmanol and epirosmanol exhibited the highest permeabilities for both formulations, particularly in the AB direction. Rosmadial and rosmanol demonstrated similar absorption values when the free and encapsulated formulations were compared. In contrast, epirosmanol and epiisorosmanol showed higher absorption values in the AB direction when encapsulated (*p*<0.001 for both). Finally, miltipolone exhibited interesting behavior: no absorption was observed in the BA direction for the free formulation, whereas no absorption was observed in the opposite direction (AB) for the encapsulated form.

Interestingly, certain facts should be noted for individual compounds. Diterpenoids, such as carnosol and its isomer, demonstrated less absorption when encapsulated, regardless of the direction analyzed. In the case of both carnosol isomers, the P_app_ values for the AB direction and for the BA direction were significantly higher for the free formulation (carnosol (*p*<0.01) and carnosol isomer (*p*<0.001)) than for the encapsulated formulation. The carnosol isomer exhibited a higher P_app_ value than carnosol in the free formulation (both directions), but no significant differences were observed for these compounds when the AB and BA directions were compared (*p* >0.05). For the encapsulated extract, no significant differences were observed between the isomers in the AB direction, but carnosol demonstrated a higher permeability than its isomer in the BA direction. These results contradict the generally accepted notion that hydrophobic diterpenes are better absorbed when encapsulated into phospholipid vesicles and thus may deserve further study.

Another interesting issue is related to the influence of certain moieties present in the compounds on absorption behavior. For carnosic and 12-methoxycarnosic acids, P_app_ values could be obtained only for the free formulation. Permeation in the AB direction was higher than in the BA direction for both compounds, but only for carnosic acid was statistically significant (*p* < 0.001). The presence of the methoxy moiety increases the permeability by almost one order of magnitude in the AB direction but reduces the permeability in the opposite direction. This result indicates that the increase in hydrophobicity attributable to the methoxy group increases absorption and concomitantly enhances retention once the compound is absorbed by reducing BA permeability. However, further studies should be undertaken to elucidate the absorption mechanism.

Considering previous data reported in the literature, it appears that the absorption observed in the present work for most of the compounds was higher than absorption values previously reported. For example, the absorption of carnosic acid and carnosol has previously been studied in a Caco-2 cell model, but no permeation was observed in certain cases [18], or longer periods of time were required to achieve permeation into the basolateral chamber [17]. No information regarding the other diterpenoids is available in the literature, either for individual compounds or complex mixtures or extracts.

#### Triterpenoids

Triterpenoids were the most abundant family in the rosemary extract (12.16% w/w) according to our previous analysis [13] and comprised six compounds (Fig 1C): augustic, betulinic, micromeric, benthamic and ursolic acids and anemosapogenin.

No absorption data for the triterpenoids could be determined based on the concentrations of the compounds in the basolateral and apical chambers when the extract was applied in the encapsulated formulation (Table 1). Augustic acid and anemosapogenin were detected only in the donor chamber but at levels below their LOQs. A similar result was obtained for ursolic, micromeric and benthamic acids; however, their concentrations could be measured in the donor chamber because they surpassed LOQs. Betulinic acid was detected both in the donor chamber and also inside the cells but was not detected in the receptor chamber. These data indicate that none of the triterpenoids are absorbed from the encapsulated formulation, independent of their chemical structure. It is likely that the high hydrophobicities of these compounds, which demonstrate high Log P values (Table 2), favor their retention in phospholipid vesicles or their association with the plasmatic membrane and other lipophilic structures inside cells.

In contrast, all triterpenoids were absorbed with low P_app_ values in the free formulation, although important differences were observed (Table 1). Ursolic acid, which showed a P_app_ values similar to that one previously reported [41]and betulinic acid were not absorbed in the AB direction and were partly retained by the Caco-2 cells. The other four triterpenoids were absorbed in the AB direction, with no significant differences (*p*>0.05) between them. All six triterpenoids were absorbed in the BA direction, also with low P_app_ values and with no significant differences between them (*p*>0.05). This indicates that the slight differences between the chemical structures of these compounds do not result in different absorption behaviors. As mentioned for the diterpenoids, encapsulation into phospholipid vesicles did not appear to be a good approach to increase triterpenoid bioavailability. As with most of the diterpenoids analyzed in the previous section, data regarding the absorption of rosemary triterpenoids are presented in this study for the first time.

The absorption of natural polyphenols has been widely studied in the Caco-2 model [36–38, 42–44]. However, scant information regarding the absorption of these compounds is available for complex mixtures such as botanical extracts. In some absorption studies, no identification of rosemary compounds was performed, and only total polyphenolic content was determined [42]. Other studies have reported the permeabilities of only a few compounds within a studied extract, employing a semiquantitative approach [17, 18, 44]. In the present study, the permeabilities of all compounds identified in the rosemary extract via HPLC ESI-UHD-Qq-TOF-MS analysis were studied in both the AB and BA directions and, additionally, were compared with an encapsulated formulation of the extract. Given that the absorptions of all compounds were analyzed in the context of the whole RE, several mechanisms of interaction, such as competition and inhibition of potentiation, between compounds may occur, particularly between compounds sharing similar chemical structures. The analyses performed in this study indicate that the permeabilities of complex botanical extracts can be fully analyzed by employing high sensitivity mass spectrometry and improved purification techniques.

Our results indicate that most compounds are scarcely absorbed, and passive diffusion is suggested to be the major mechanism of absorption for most compounds, as the AB and BA directions both yielded similar results. Further mechanistic studies must be carried out to elucidate this mechanism. In contrast, we confirmed that contrary to previous observations for other compounds, the use of liposomes to encapsulate RE compounds is not a good approach to improve their permeability because P_app_ values were reduced or negligible for the majority of the compounds. According to Fick’s Law, absorption depends on the permeability/diffusion constant and concentration of the compound. If the solubility of a compound demonstrating low absorption is increased due to encapsulation into liposomes and its concentration is enhanced in the donor chamber, an increase in the absorption may occur to compensate for its reduced absorption, as reported for other hydrophobic natural compounds [45]. This does not appear to be the case for the diterpenoids and triterpenoids in RE, but further studies must be carried out to clarify these observations.

### Biopharmaceutical classification

The Biopharmaceutical Classification System (BCS) was developed by Amidon and coworkers twenty years ago to classify drugs for a waiver of *in vivo* bioequivalence studies [46]. This system classifies drugs into four categories based on their intestinal permeability and solubility (see S1 Table), and currently this system is accepted worldwide by researchers and governmental institutions such as the FDA and EMA. In addition to its regulatory use, BCS is a tool for drug development because it helps identify the limiting factors in the absorption process [47]. In this work, the 24 compounds identified from RE were classified individually according to this system to obtain relevant information regarding their absorbed oral fraction. For this purpose, only data derived from the free extract permeation were used.

#### Permeability values

Log P values were obtained for all compounds using ChemDraw software® Ultra version 8.0 (CambridgeSoft Corp. USA) and are shown in Table 2. The Log P value is a widely accepted parameter to express the lipophilicities of biological drugs in medicinal and agro chemistry. According to BCS, compounds are divided into high permeability (Class I: high permeability, high solubility; Class II: high permeability, low solubility) or low permeability (Class III: low permeability, high solubility; Class IV: low permeability, low solubility) categories by comparing with the Log P value (2.18) of the standard metoprolol, which is the reference compound employed for this purpose [48, 49]. Based on this parameter, the permeabilities of all compounds derived from RE were tabulated as high or low (Table 2). Moreover, the P_app_ value of this reference drug (0.6 x 10^−4^ cm/s) was also available, which allowed us to establish another classification for the permeabilities of RE compounds based on this parameter, using the P_app_ values of the compounds obtained in the AB direction only for the free formulations (Table 2). Therefore, the Log P and P_app_ values were used in Table 2 to classify compounds as high- or low-solubility drugs for permeability, providing two different classifications based on the partition coefficient and the intestinal permeability.

### Solubility values

Together with permeability, water solubility is another parameter that is required to classify drugs or compounds according BCS. For this purpose, the dose number value, D_0_, must be obtained from the following equation:

$$D_{0}=\frac{maximum\;administered\;dose}{S_{w}\cdot 250}$$

where S_w_ is the minimum solubility of each compound in water (in the pH range from 1.2 to 6.8) and the 250 value indicates the volume of a glass of water. A value of D_0_ ≤ 1 indicates that the maximum administered dose can be dissolved in a glass of water; thus, the compound is classified as a high-solubility drug. Unfortunately, natural compounds such as polyphenols and terpenoids exhibit poor solubility. No experimental data regarding the solubilities of the 24 compounds identified from RE in this study are available. This problem was overcome using Marvin Suite software (version 16.1.11.0, ChemAxon Ltd.), which provides several physicochemical properties, including a prediction of water solubility (S_w_). Table 2 includes the S_w_ values for all 24 compounds.

**Fig 2:**
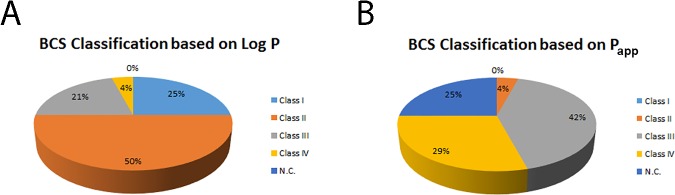
BCS classification. Plots show the BCS classification of all compounds in RE using the 10 mg dose scenario according LogP (A) y P_aap_ (B) values.

As no information was available regarding the maximum doses of these compounds, two putative scenarios were selected to simulate a high-activity drug, dosed as a 10-mg tablet, or a low-activity drug, dosed as a 100-mg tablet. Table 2 shows the results for the high-activity drug (10-mg dose), and S2 Table includes results from the second option (100-mg dose). In the present study, all compounds were classified for BCS by considering the experimental data for absorption (P_app_) and the calculated Log P value (Table 2). According to the Log P value, most compounds belong to the high-permeability classes (I and II), with class II being the most numerous (Fig 2A), except in the case of the flavonoids. However, when experimental P_app_ values were used for classification, the results changed dramatically, and low-permeability classes (III and IV) became the most abundant (Fig 2B). This classification was nearly independent of the solubility scenario and considered dose (compare Fig 2 and S1 Fig). Moreover, the different families of compounds also exhibited different behaviors in terms of BCS classification. Most flavones in RE were classified as class III (low permeability, high solubility) and class IV (low permeability, low solubility) with the exception of apigenin, for which no experimental P_app_ could be determined and, consequently, no BCS classification was proposed. This result was in agreement with recently reported data and reinforces the notion that the limited bioavailabilities of most polyphenols may be improved by strategies focused on increasing drug solubilities and dissolution rates in the gastrointestinal fluid [50]. The phenylpropanoid derivatives shogaol and shogaol isomer (gingerol-like compounds) were classified as class II (high permeability, low solubility), which is in agreement with the presence of a long saturated carbon chain in their structure that confers a certain degree of hydrophobicity to these compounds.

Conversely, most terpenoids demonstrated different classes depending on the use of Log P or P_app_ for their classification (see Table 2). Both diterpenoids and triterpenoids were primarily classified as class II (high permeability, low solubility) or class IV (low permeability, low solubility) when Log P or P_app_ was utilized, respectively, for the classification. A regulatory classification of any compound must be based on experimental data, but the Log P approach has previously been used for screening purposes [48, 49]. Based on our results, we confirm that important differences may arise when experimental P_app_ values are used to classify compounds versus Log P approximation. It can be concluded that the P_app_ value, when available, is a more appropriate alternative to establish BCS classification because this value is derived from data determined in a living cellular model, which provides results with greater biological significance than those obtained from calculations based on physicochemical characteristics.

It must be noted that in this study, S_w_ was obtained for the individual compounds. However, the solubilities of the compounds in the complex RE mixture may be affected by the presence of other compounds, and this will exert an undeniable impact on the absorption of the compound. This feature deserves further investigation.

In conclusion, BCS classification of the 24 compounds present in the RE extract was achieved by considering the aforementioned limitations and using experimental P_app_ rather than Log P when available. This revealed that most of the compounds in RE were classified as classes III and IV. Based on this, RE enriched in diterpenes and triterpenes should also be classified as class III or IV (low permeability), which is in agreement with the low bioavailabilities of rosemary compounds reported in the literature. However, this statement should be interpreted with caution due to the complexity of the extract, and further research may be required before a final classification is given. The results obtained here represent a significant contribution to our knowledge of the oral absorptions and bioavailabilities of rosemary compounds and their biopharmaceutical classifications and will aid in the development of delivery strategies to improve solubility and bioavailability. In addition, as rosemary compounds have demonstrated their influence in microbioma in previous studies [51–53] this point must be also addressed in future studies.

## Conclusions

Rosemary is widely used as a medicinal herb and to season and preserve food, and its bioactive compounds (terpenoids and polyphenols) possess demonstrated potential health benefits related to chronic human diseases. Although most studies have been performed in cell models, recent evidence for such effects is emerging in animal models. However, only a few studies have explored the intestinal absorption of these compounds to determine their permeability and the metabolites responsible for such effects. In this study, we provide data regarding the permeabilities of 24 compounds derived from a rosemary extract in Caco-2 cell monolayers (flavonoids, diterpenes, triterpenes and phenylpropanoids) by comparing the extract in both free and encapsulated form. Flavonoids demonstrated a passive diffusion transport mechanism, with cirsimaritin and genkwanin having the highest permeation values. Among the diterpenes, carnosic acid, rosmanol and its isomers epiisorosmanol and epirosmanol exhibited the highest permeability values. Triterpenoids exhibited lower permeability values than diterpenes and flavonoids. Most compounds demonstrated poor or negligible permeation when RE was incorporated into phospholipid vesicles. For some of the aforementioned compounds, this study is the first report of their permeability. In this study, we also performed biopharmaceutical classification (BCS) for all the compounds based on their permeability and solubility data for bioequivalence purposes, which may represent a basis for future studies focused on the development of rosemary-based nutraceuticals or drugs. Most of the RE compounds were classified as classes III and IV (low permeability); therefore, RE should also be classified into this category.

## Supporting information

S1 Fig BCS classification. Plots show the BCS classification of all compounds in RE using the 100 mg dose scenario according LogP (A) y P_aap_ (B) values.
(DOCX)

S1 Table BCS classification. (DOCX)

S2 Table Chemical family, physicochemical and permeation data and BCS classification. Chemical family, physicochemical and permeation data and BCS classification for all the compounds of RE studied in the absorption assay in the free form and considering 100 mg dose scenario.
(DOCX)

S3 Table TEER values. Trans-epithelial electrical resistance (TEER) obtained in Ω.cm^2^units by using an epithelial voltohmmeter (Millicell-ERS®). Each weel (6 wells were used for each condition) was measured three independents times before sample addition (initial value) and at the end of the incubation time (final value).
(DOCX)
