# Supplementary material for: Evaluation of the intestinal permeability of rosemary (Rosmarinus officinalis L.) extract polyphenols and terpenoids in Caco-2 cell monolayers
Source: PLoS One. 2017 Feb 24;12(2):e0172063. doi: 10.1371/journal.pone.0172063 (PMC5325326; doi:10.1371/journal.pone.0172063)
Supplement: S1 Fig — Plots show the BCS classification of all compounds in RE using the 100 mg dose scenario according LogP (A) y Paap (B) values. (DOCX) [file pone.0172063.s001.docx]

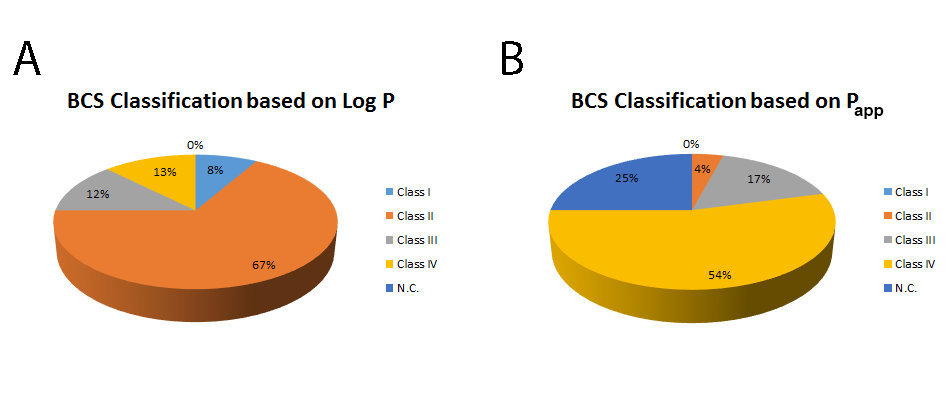


**S1 Fig**. **BCS classification**. Plots show the BCS classification of all compounds in RE using the 100 mg dose scenario according LogP (A) y P_aap_ (B) values.
