# Supplementary material for: Evaluation of the intestinal permeability of rosemary (Rosmarinus officinalis L.) extract polyphenols and terpenoids in Caco-2 cell monolayers
Source: PLoS One. 2017 Feb 24;12(2):e0172063. doi: 10.1371/journal.pone.0172063 (PMC5325326; doi:10.1371/journal.pone.0172063)
Supplement: S1 Table — (DOCX) [file pone.0172063.s002.docx]

**S1 Table. BCS classification.**

|  | **High solubility** | **Low solubility** |
| --- | --- | --- |
| **High**  **permeability** | Class I | Clas II |
|  | High Permeability | High permeability |
|  | High solubility | Low solubility |
| **Low permeability** | Class III | Class IV |
|  | Low permeability | Low permeability |
|  | High solubility | Low solubility |

The Biopharmaceutics Classification System.
