# Supplementary material for: Evaluation of the intestinal permeability of rosemary (Rosmarinus officinalis L.) extract polyphenols and terpenoids in Caco-2 cell monolayers
Source: PLoS One. 2017 Feb 24;12(2):e0172063. doi: 10.1371/journal.pone.0172063 (PMC5325326; doi:10.1371/journal.pone.0172063)
Supplement: S2 Table — Chemical family, physicochemical and permeation data and BCS classification for all the compounds of RE studied in the absorption assay in the free form and considering 100 mg dose scenario. (DOCX) [file pone.0172063.s003.docx]

**S2 table. Chemical family, physicochemical and permeation data and BCS classification.**

| **Compound** | **Family** | **D (M)** | **S (mg/mL)** | **Vs** | **D_0_** | **Solubility** | **Log P** | **Permeability** | **BCS Class** |
| --- | --- | --- | --- | --- | --- | --- | --- | --- | --- |
|  |  |  |  |  |  |  |  | **Based on Log P** | **Based on Log P** |
|  |  |  |  |  |  |  |  | **Based on P_app_** | **Based on P_app_** |
| Apigenin | Flavonoid | 0.37 | 4.46E-03 | 82.84 | 0.33 | High | 1.90 | Low | III |
|  |  |  |  |  |  |  |  | N.C. | N.C. |
| Cirsimaritin | Flavonoid | 0.32 | 2.57E-04 | 1237.85 | 4.95 | Low | 2.04 | Low | IV |
|  |  |  |  |  |  |  |  | Low | IV |
| Diosmetin | Flavonoid | 0.33 | 4.27E-03 | 78.07 | 0.31 | High | 1.78 | Low | III |
|  |  |  |  |  |  |  |  | Low | III |
| Hispidulin | Flavonoid | 0.33 | 1.58E-03 | 210.13 | 0.84 | High | 1.78 | Low | III |
|  |  |  |  |  |  |  |  | Low | III |
| Genkwanin | Flavonoid | 0.35 | 4.17E-04 | 843.87 | 3.38 | Low | 2.17 | Low | IV |
|  |  |  |  |  |  |  |  | Low | IV |
| Carnosol | Diterpene | 0.30 | 2.57E-05 | 11774.31 | 47.10 | Low | 4.58 | High | II |
|  |  |  |  |  |  |  |  | Low | IV |
| Carnosol Isomer | Diterpene | 0.30 | 2.57E-05 | 11774.31 | 47.10 | Low | 4.58 | High | II |
|  |  |  |  |  |  |  |  | Low | IV |
| Carnosic acid | Diterpene | 0.30 | 1.51E-02 | 19.87 | 0.08 | High | 5.14 | High | I |
|  |  |  |  |  |  |  |  | Low | III |
| 12-methoxycarnosic acid | Diterpene | 0.29 | 5.50E-03 | 52.52 | 0.21 | High | 5.40 | High | I |
|  |  |  |  |  |  |  |  | Low | III |
| Rosmadial | Diterpene | 0.29 | 9.12E-05 | 3183.71 | 12.73 | Low | 3.74 | High | II |
|  |  |  |  |  |  |  |  | Low | IV |
| Rosmanol | Diterpene | 0.29 | 5.75E-05 | 5016.48 | 20.07 | Low | 3.70 | High | II |
|  |  |  |  |  |  |  |  | Low | IV |
| Epirosmanol | Diterpene | 0.29 | 5.75E-05 | 5016.48 | 20.07 | Low | 3.70 | High | II |
|  |  |  |  |  |  |  |  | Low | IV |
| Epiisorosmanol | Diterpene | 0.29 | 5.75E-05 | 5016.48 | 20.07 | Low | 3.70 | High | II |
|  |  |  |  |  |  |  |  | High | II |
| Miltipolone | Diterpene | 0.33 | 3.55E-05 | 9382.33 | 37.53 | Low | 1.02 | Low | IV |
|  |  |  |  |  |  |  |  | Low | IV |
| Hinokione | Diterpene | 0.33 | 2.19E-05 | 15214.17 | 60.86 | Low | 5.85 | High | II |
|  |  |  |  |  |  |  |  | N.C. | N.C. |
| Rosmaridiphenol | Diterpene | 0.32 | 1.55E-06 | 204040.77 | 816.16 | Low | 4.89 | High | II |
|  |  |  |  |  |  |  |  | Low | IV |
| Augustic acid | Triterpene | 0.21 | 1.15E-04 | 1842.53 | 7.37 | Low | 6.52 | High | II |
|  |  |  |  |  |  |  |  | Low | IV |
| Betulinic acid | Triterpene | 0.22 | 2.04E-05 | 10724.27 | 42.90 | Low | 7.38 | High | II |
|  |  |  |  |  |  |  |  | N.C. | N.C. |
| Anemosapogenin | Triterpene | 0.21 | 1.20E-04 | 1759.60 | 7.04 | Low | 6.32 | High | II |
|  |  |  |  |  |  |  |  | Low | IV |
| Micromeric acid | Triterpene | 0.22 | 1.23E-04 | 1787.68 | 7.15 | Low | 6.91 | High | II |
|  |  |  |  |  |  |  |  | Low | IV |
| Benthamic acid | Triterpene | 0.21 | 5.89E-04 | 359.26 | 1.44 | Low | 6.13 | High | II |
|  |  |  |  |  |  |  |  | Low | IV |
| Ursolic acid | Triterpene | 0.22 | 1.51E-05 | 14466.64 | 57.87 | Low | 7.33 | High | II |
|  |  |  |  |  |  |  |  | N.C. | N.C. |
| [9]-Shogaol | Phenylpropanoid | 0.31 | 1.29E-06 | 243758.85 | 975.04 | Low | 5.26 | High | II |
|  |  |  |  |  |  |  |  | N.C. | N.C. |
| [9]-Shogaol Isomer | Phenylpropanoid | 0.31 | 1.29E-06 | 243758.85 | 975.04 | Low | 5.26 | High | II |
|  |  |  |  |  |  |  |  | N.C. | N.C. |
|  |  |  |  |  |  |  |  |  |  |

Chemical family, physicochemical and permeation data and BCS classification for all the compounds of RE studied in the absorption assay in the free form and considering 100 mg dose scenario.
