# Supplementary material for: Evaluation of the intestinal permeability of rosemary (Rosmarinus officinalis L.) extract polyphenols and terpenoids in Caco-2 cell monolayers
Source: PLoS One. 2017 Feb 24;12(2):e0172063. doi: 10.1371/journal.pone.0172063 (PMC5325326; doi:10.1371/journal.pone.0172063)
Supplement: S3 Table — Trans-epithelial electrical resistance (TEER) obtained in Ω.cm2units by using an epithelial voltohmmeter (Millicell-ERS®). Each weel (6 wells were used for each condition) was measured three independents times before sample addition (initial value) and at the end of the incubation time (final value). (DOCX) [file pone.0172063.s004.docx]

**S3 Table: TEER values.**

|  |  | TEER values (Ohm·cm^2^) | |
| --- | --- | --- | --- |
|  |  | Mean | SD |
| RE in liposomes | Initial | 212,9 | 28,8 |
|  | Final | 218,4 | 23,0 |
| RE | Initial | 214,3 | 24,2 |
|  | Final | 200,8 | 17,0 |
|  |  |  |  |

Trans-epithelial electrical resistance (TEER) obtained in Ω.cm^2^units by using an epithelial voltohmmeter (Millicell-ERS®). Each weel (6 wells were used for each condition) was measured three independents times before sample addition (initial value) and at the end of the incubation time (final value).
